# The Efficacy and Safety of Programmed Death-1 and Programmed Death Ligand 1 Inhibitors for the Treatment of Hepatocellular Carcinoma: A Systematic Review and Meta-Analysis

**DOI:** 10.3389/fonc.2021.626984

**Published:** 2021-03-23

**Authors:** Shukang He, Weichao Jiang, Kai Fan, Xiaobei Wang

**Affiliations:** Department of Clinical Laboratory, Union Hospital, Tongji Medical College, Huazhong University of Science and Technology, Wuhan, China

**Keywords:** hepatocellular carcinoma, immune checkpoint inhibitors, PD-1/PD-L1 inhibitors, immunotherapy, meta-analysis

## Abstract

**Background:**

Hepatocellular carcinoma (HCC) is often diagnosed at an advanced stage where only systemic treatment can be offered. The emergence of immune checkpoint inhibitors (ICIs) provides hope for the treatment of HCC. In this study, we performed a meta-analysis to provide evidence for the efficacy and safety of ICIs in the treatment of HCC.

**Methods:**

The following databases and websites were searched: Embase, PubMed, Cochrane Library and ClinicalTrials.gov. The primary endpoints were response rate (RR), disease control rate (DCR), progression-free survival (PFS) and overall survival (OS).

**Results:**

Finally, twelve studies were included in this meta-analysis. When the corresponding outcome indicators and their 95% confidence intervals (CIs) were pooled directly, the overall RR, DCR, PFS and OS were 0.17 (0.15-0.19, I^2^ = 56.2%, P=0.009), 0.58 (0.55-0.61, I^2^ = 75.9%, P<0.001), 3.27 months (2.99-3.55, I^2^ = 73.0%, P=0.001), 11.73 months (10.79-12.67, I^2^ = 90.3%, P<0.001). Compared to the control group, treatment with ICIs significantly improved RR, PFS and OS, the OR and HRs were 3.11 (2.17-4.44, P<0.001), 0.852 (0.745-0.974, P=0.019) and 0.790 (0.685-0.911, P=0.001), respectively. However, no significant improvement in DCR was found in ICIs treatment in this meta-analysis.

**Conclusion:**

HCC patients would benefit from ICIs treatment, however, more studies are needed in the future to provide more useful evidence for the treatment of HCC by programmed death-1 (PD-1) or programmed death ligand 1 (PD-L1) inhibitors.

## Introduction

Primary liver cancer is the sixth most common tumor in the world and the fourth leading cause of cancer-related death, of which 75% to 85% are hepatocellular carcinoma (HCC) (1). Chronic infection with hepatitis C virus (HCV) or hepatitis B virus (HBV) is the leading cause of hepatocellular carcinoma (2). Additionally, HCC is often diagnosed at an advanced stage where only systemic treatment can be offered (3). Although many measures have been taken, the incidence of HCC has increased during the last decade globally and increases progressively with advancing age in all populations (4).

For a long time, there has been a lack of effective systemic therapy for advanced HCC. In the past decade, sorafenib was the only approved first-line agent for patients with unresectable or metastatic hepatocellular carcinoma (3, 5, 6). However, the benefits of sorafenib as the first-line standard treatment were limited. In the global and Asian phase III studies, compared with the placebo group, the median overall survival (OS) of patients in the sorafenib group was only extended by about 2 months, and the objective response rate (ORR) was only 2%-3.3%, and it often causes adverse events (7, 8). Targeted agents currently used in patients with HCC, such as sorafenib, regorafenib, and lenvatinib, are multikinase inhibitors, which have lower response rates and higher therapeutic resistance than targeted therapy agents in other cancers (9).

The emergence of immune checkpoint inhibitors (ICIs) provides hope for the treatment of hepatocellular carcinoma. ICIs are designed to block immunosuppressive receptors expressed on the surface of T lymphocytes such as cytotoxic T-lymphocyte-associated antigen 4, programmed death receptor-1 (PD-1), and the programmed death-ligand 1 (PD-L1) expressed on tumor cells and tumor-infiltrating immune cells (10). At present, immunotherapy, together with surgery, radiotherapy, chemotherapy and targeted therapy, has become the mainstay of the treatment of malignant tumors. Therapeutic monoclonal antibodies targeting PD-1 or PD-L1 have demonstrated notable clinical efficacy in the treatment of various advanced cancers, including non-small-cell lung cancer (NSCLC), melanoma, hepatocellular carcinoma et al. (11).

In this study, the existing literature on the treatment of HCC with PD-1 or PD-L1 inhibitors was retrieved, and a meta-analysis was conducted to provide evidence for the efficacy and safety of PD-1/PD-L1 inhibitors in the treatment of HCC.

## Materials and Methods

We conducted this meta-analysis according to the Preferred Reporting Items for Systematic Review and Meta-Analysis guidelines (PRISMA) (12).

### Data Sources and Searches

The following databases and websites were searched: Embase, PubMed, Cochrane Library and ClinicalTrials.gov. Key words used were: hepatocellular carcinoma; PD-1/PD-L1 inhibitors, nivolumab, pembrolizumab, camrelizumab, tislelizumab, atezolizumab. The time limit was from the establishing of the databases to October 2020. References in the eligible articles would also be searched when necessary.

### Study Selection

Inclusion criteria: (1) Study design: Randomized controlled trials (RCTs), cohort studies or single-arm studies about the treatment of HCC with PD-1 or PD-L1 inhibitors. (2) Population: patients with HCC. (3) Intervention and comparison: PD-1 or PD-L1 inhibitors were compared with placebo or other non-ICI drugs for HCC, such as sorafenib. (4) Outcomes: response rate [RR, defined as defined as patients with complete or partial response (9)], disease control rate [DCR, defined as patients with complete response, partial response, or stable disease (9)], progression-free survival [PFS, median, defined as the time from the date of first checkpoint inhibitor administration until radiological disease progression or death, whatever came first (3)] and overall survival [OS, median, defined as the time from the date of first checkpoint inhibitor administration until death (3)]. Exclusion criteria: (1) Duplicated articles. (2) Articles with too small sample size to extract data. (3) Articles that did not provide outcomes needed. (4) Articles about the combination of ICIs with other treatments for HCC. (5) Articles in other languages than English.

### Data Extraction and Quality Assessment

Two independent investigators screened the articles and extracted the data. If there was any disagreement, it would be resolved through discussion between the two investigators or by a third investigator. The data extracted were: publication year, countries, trial names, study registration no., inhibitors used, number of patients and their median ages (years), RR, DCR, PFS and OS.

### Statistical Analysis

The data was analyzed by Stata 14.0 (StataCorp), Excel (Microsoft office 2016) and SPSS 21.0 (IBM SPSS Statistics). I^2^ statistic was used to evaluate the heterogeneity among studies. If I^2^<50% or P>0.10, then the heterogeneity was considered to be low and fixed-effects model was applied. Otherwise, the random-effects model was applied. For the single-arm study, outcomes were pooled to get overall RR, DCR, PFS and OS. Hazard ratios (HRs) were used to analyze the PFS and OS and odd ratios (ORs) were used to analyze the RR and DCR. P<0.05 indicated that the results were statistically significant.

## Results

### Search Results and Study Quality Assessment

**Figure 1:**
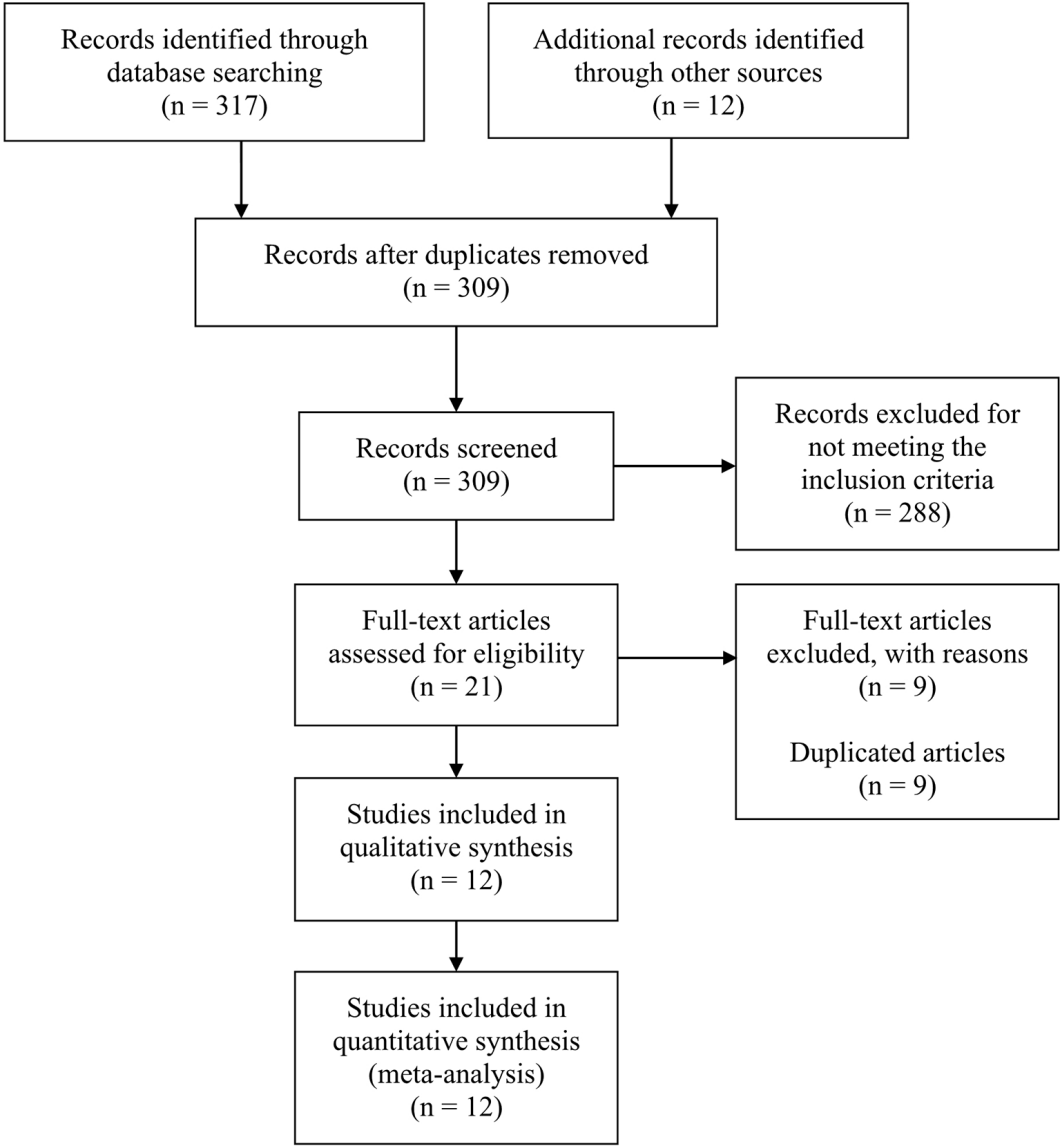
Flow chart of study selection.

This meta-analysis searched a total of 317 studies, and finally 12 studies were included, among which 8 were single-arm studies, 2 were RCTs and 2 were retrospective cohort studies. **Figure 1** displayed the flow chart of study selection. The PD-1 or PD-L1 inhibitors involved in these studies were nivolumab (7 studies), pembrolizumab (3 studies), camrelizumab (1 study), cemiplimab (1 study) and tislelizumab (1 study). The characteristics of included studies were shown in **Table 1**. **Table 2** displayed the characteristics of the patients in the studies included in this meta-analysis.

**Table 1:** Characteristics of included studies.

Study	Year	Country	Trial name	Study registration no.	Inhibitor	Number of patients	Median age (years)	Response rate	Disease control rate	Progression-free survival (months)	Overall survival (months)
El-Khoueiry et al. (2)	2017	Global	CheckMate 040	NCT01658878	Nivolumab	262	62 (Escalation phase) 64 (Expansion phase)	0.20 (0.15-0.26) (Escalation phase) 0.15 (0.60-0.28) (Expansion phase)	0.64 (0.58-0.71) (Escalation phase) 0.58 (0.43-0.72) (Expansion phase)	4.0 (2.9-5.4) (Escalation phase) 3.4 (1.6-6.9) (Expansion phase)	N/A (Escalation phase) 15.0 (9.6-20.2) (Expansion phase)
Feng et al. (6)	2017	China	N/A	N/A	Nivolumab	11	55	0.64 (0.30-0.98)	0.82 (0.55-1.09)	N/A	N/A
Zhu et al. (13)	2018	Global	KEYNOTE-224	NCT02702414	Pembrolizumab	104	68	0.17 (0.11-0.26)	0.62 (0.52-0.71)	4.9 (3.4-7.2)	12.9 (9.7-15.5)
Pishvaian et al. (14)	2018	Global	N/A	NCT02383212	Cemiplimab	26	65	N/A	0.73 (0.55-0.91)	3.7 (2.3-9.1)	N/A
Deva et al. (15)	2018	Global	N/A	NCT02407990	Tislelizumab	207	N/A	0.12 (0.05-0.25)	0.51 (0.36-0.66)	N/A	N/A
Finkelmeier et al. (16)	2019	Germany	N/A	N/A	Nivolumab	34	65	0.12 (0.003-0.23)	0.35 (0.15-0.55)	N/A	3.6 (0-7.1)
Finn et al. (17)	2019	Global	KEYNOTE-240	NCT02702401	Pembrolizumab	413	67 (Pembrolizumab) 65 (Placebo)	0.18 (0.14-0.23) (Pembrolizumab) 0.04 (0.016-0.094) (Placebo)	0.62 (0.56-0.68) (Pembrolizumab) 0.53 (0.45-0.62) (Placebo)	3.0 (2.8-4.1) (Pembrolizumab) 2.8 (1.6-3.0) (Placebo)	13.9 (11.6-16.0) (Pembrolizumab) 10.6 (8.3-13.5) (Placebo)
Yau et al. (18)	2019	Global	CheckMate 459	NCT02576509	Nivolumab	743	64 (Nivolumab) 65 (Sorafenib)	0.15 (0.12-0.19) (Nivolumab) 0.07 (0.046-0.10) (Sorafenib)	N/A (Nivolumab) N/A (Sorafenib)	3.7 (3.1-3.9) (Nivolumab) 3.8 (3.7-4.5) (Sorafenib)	16.4 (13.9-18.4) (Nivolumab) 14.7 (11.9-17.2) (Sorafenib)
Scheiner et al. (3)	2019	Austria/Germany	N/A	N/A	Nivolumab/Pembrolizumab	65	65	0.12 (0.04-0.21)	0.49 (0.37-0.62)	4.6 (3.0‐6.2)	11.0 (8.2‐13.8)
Qin et al. (5)	2020	China	N/A	NCT02989922	Camrelizumab	217	49	0.15 (0.10-0.20)	0.44 (0.38-0.51)	2.1 (2.0-3.2)	13.8 (11·5-16·6)
Choi et al. (9)	2020	Korea	N/A	N/A	Nivolumab	373	59 (Regorafenib) 57 (Nivolumab)	0.04 (0.01-0.07) (Regorafenib) 0.13 (0.08-0.19) (Nivolumab)	0.47 (0.40-0.53) (Regorafenib) 0.39 (0.31-0.47) (Nivolumab)	12.0 (9.1-13.3) weeks (Regorafenib) 7.1 (6.3-10.1) weeks (Nivolumab)	30.9 (28.9-35.6) weeks (Regorafenib) 32.6 (21.7-42.9) weeks (Nivolumab)
Lee et al. (19)	2020	Korea	N/A	N/A	Nivolumab	150	62 (Regorafenib) 61 (Nivolumab)	0.06 (0.01-0.11) (Regorafenib) 0.17 (0.06-0.28) (Nivolumab)	0.47 (0.37-0.57) (Regorafenib) 0.50 (0.35-0.65) (Nivolumab)	N/A (Regorafenib) N/A (Nivolumab)	6.9 (3.0-10.8) (Regorafenib) 5.9 (3.7-8.1) (Nivolumab)

N/A, not available.

**Table 2:** Characteristics of the patients in the included studies.

Characteristics		Total
≥65 years		502
Sex	Male	1788
	Female	361
Race	White	612
	Asian	465
	Black	16
	Other	15
ECOG performance status	0	476
	1	632
	2	7
Extrahepatic metastases		985
Vascular invasion		212
Child–Pugh score	A	1261
	B	128
	C	6
Baseline AFP	>200 ng/mL	441
	≤200 ng/mL	290
Previous treatment	Surgical resection	188
	Systemic therapy	283
	Sorafenib	676
	Loco-regional (TACE/SIRT/radiation)	45
BCLC stage	B	157
	C	977
Alcohol use		344
HBV		640
HCV		175

Some characteristics were not available in some studies.

AFP, a-fetoprotein; ECOG, Eastern Cooperative Oncology Group; BCLC stage, Barcelona Clinic Liver Cancer stage. HBV, hepatitis B virus. HCV, hepatitis C virus.

**Figure 2:**
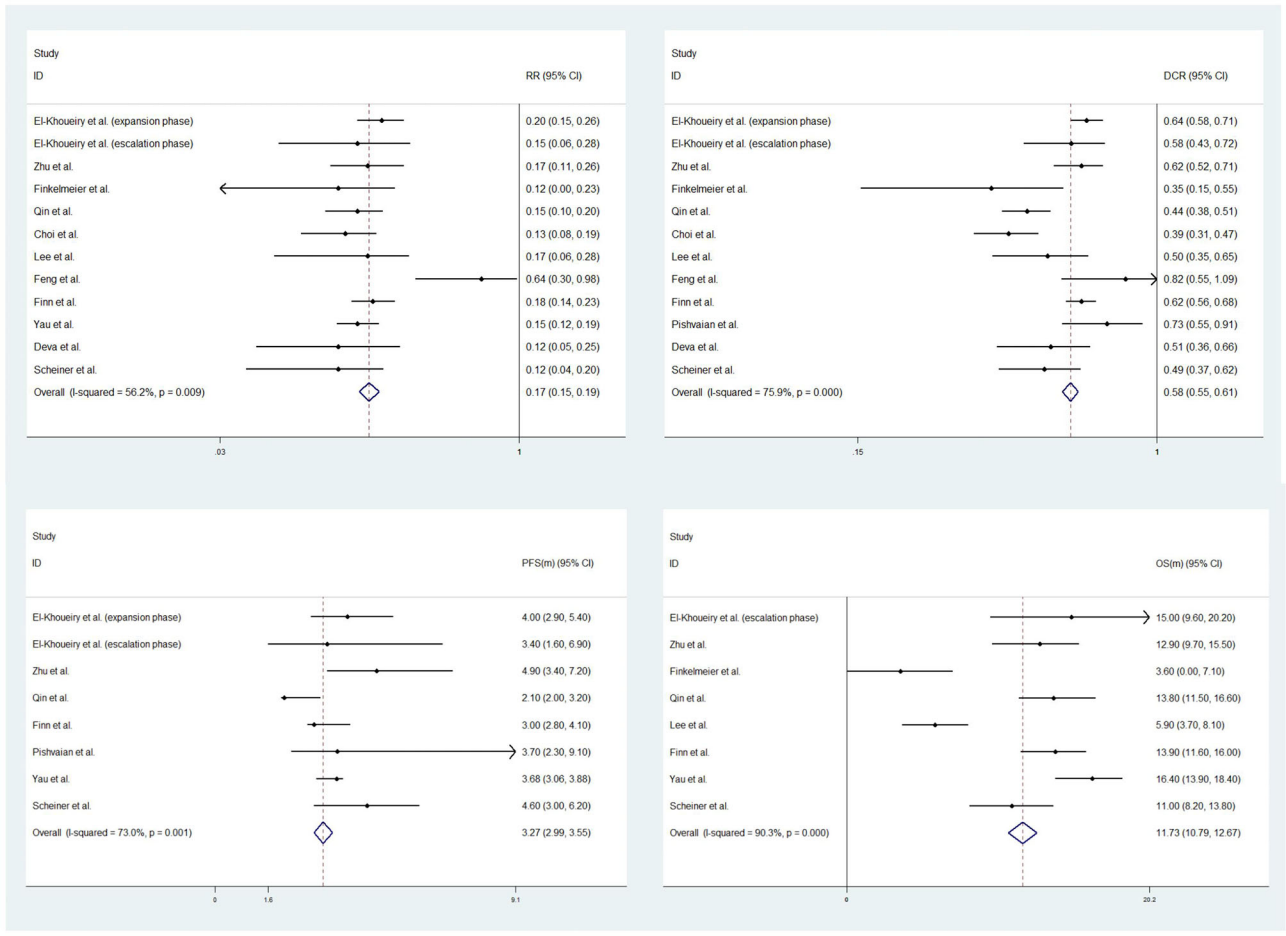
Forest plots of response rates (RR) (upper left), disease control rates (DCR) (upper right), progression-free survival (PFS) (low left) and overall survival (OS) (low right).

This meta-analysis focused on 4 outcomes: response rates (RR), disease control rates (DCR), progression-free survival (PFS) and overall survival (OS). For single-arm studies, the corresponding outcome indicators and their 95% confidence intervals (CIs) were pooled directly due to the lack of control group data. Data from RCTs or cohort studies would also be pooled with single-arm studies if they reported the same outcome indicators. There were 11, 11, 7, 8 studies in this meta-analysis reporting corresponding RR, DCR, PFS, OS data and their 95% CIs, respectively. The overall RR, DCR, PFS and OS were 0.17 (0.15-0.19, I^2 =^ 56.2%, P=0.009), 0.58 (0.55-0.61, I^2 =^ 75.9%, P<0.001), 3.27 months (2.99-3.56, I^2 =^ 73.0%, P=0.001), 11.73 months (10.79-12.67, I^2 =^ 90.3%, P<0.001). See **Figure 2** for details.

**Figure 3:**
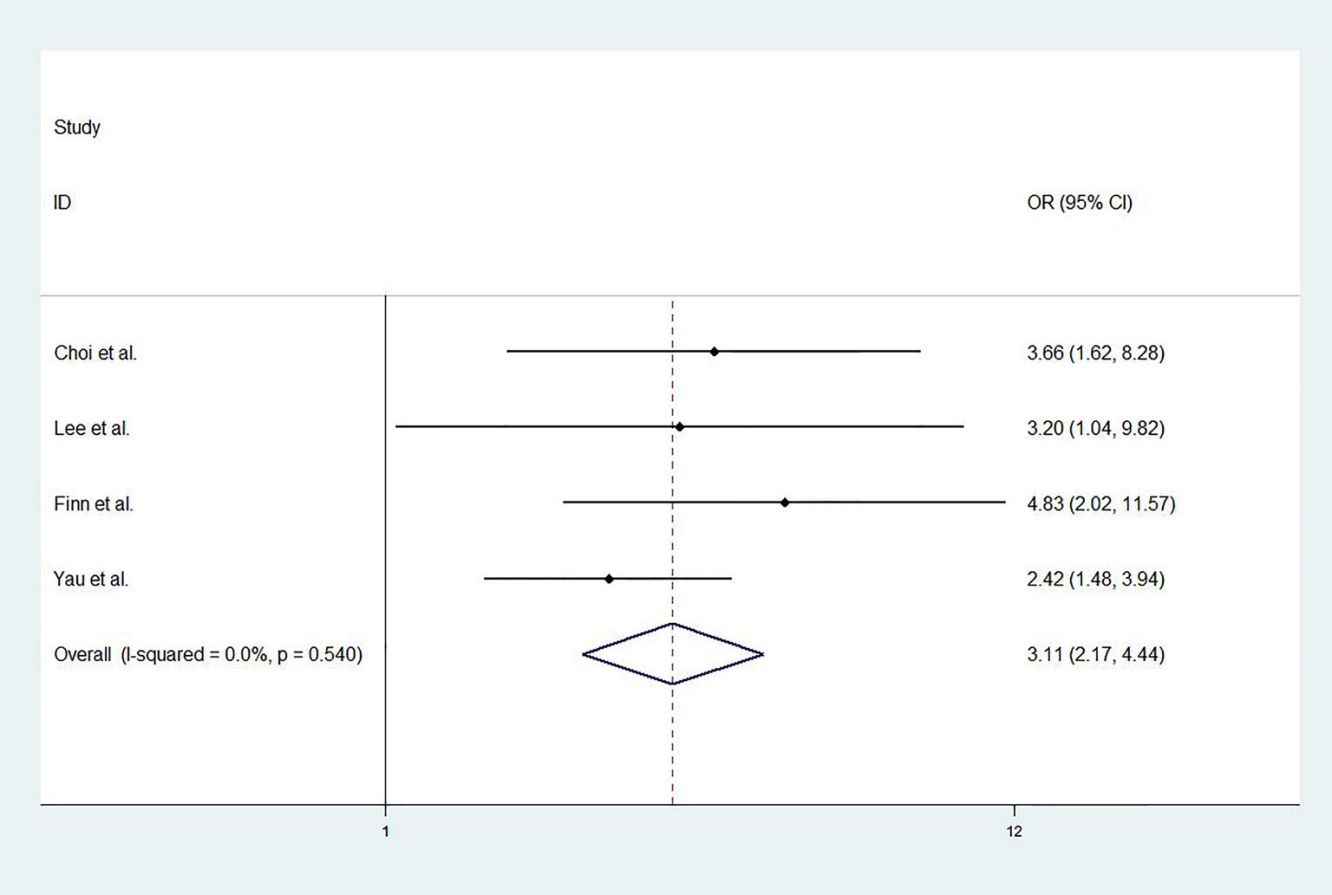
The forest plot of response rates (RR).

For studies with control group data (RCTs or cohort studies), data from the experimental and control groups were analyzed. For RR and DCR, the corresponding ORs were 3.11 (2.17-4.44, P<0.001) and 1.05 (0.80-1.37, P=0.731), and I^2^ were 0.0% (P=0.540) and 59.1% (P=0.087). For PFS and OS, HRs were 0.852 (0.745-0.974, P=0.019) and 0.790 (0.685-0.911, P=0.001), respectively. The I^2^ were 68.7% (P=0.074) and 72.5% (P=0.027). **Figures 3** **–** **6** displayed the forest plots of RR, DCR, PFS and OS. The treatment-related adverse events in the included studies were shown in **Table 3**.

**Figure 4:**
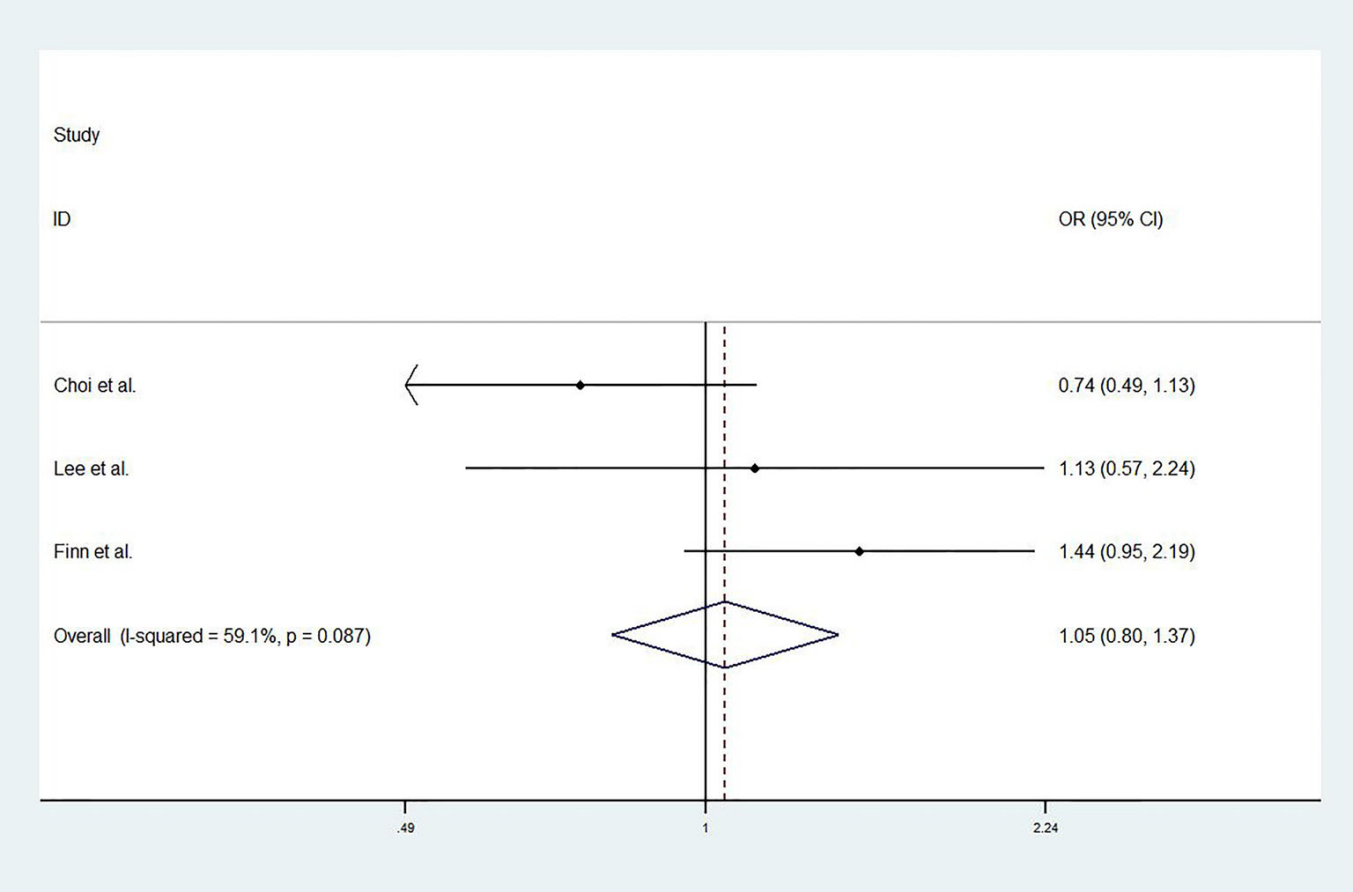
The forest plot of disease control rates (DCR).

**Figure 5:**
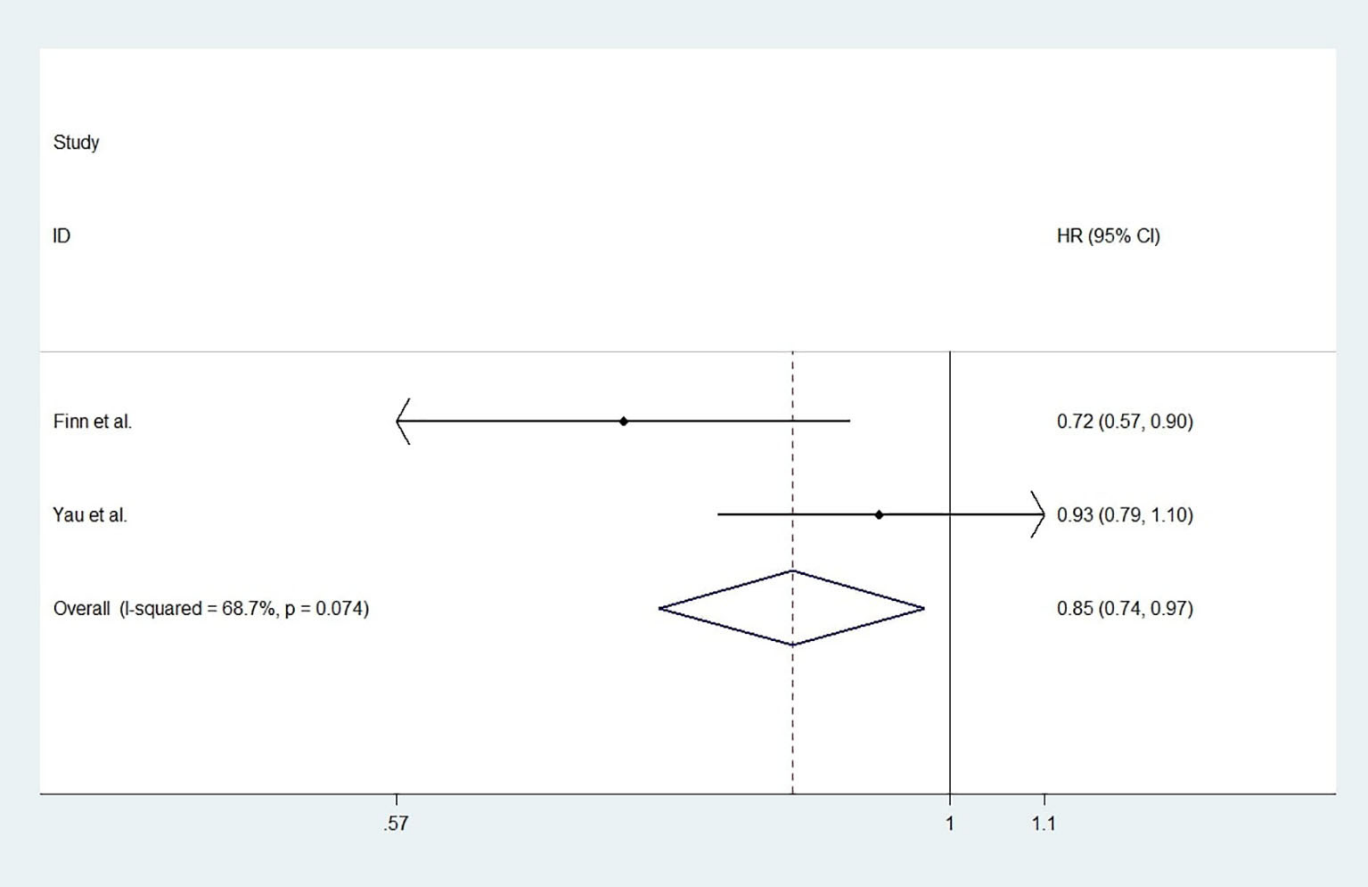
The forest plot of progression-free survival (PFS).

**Figure 6:**
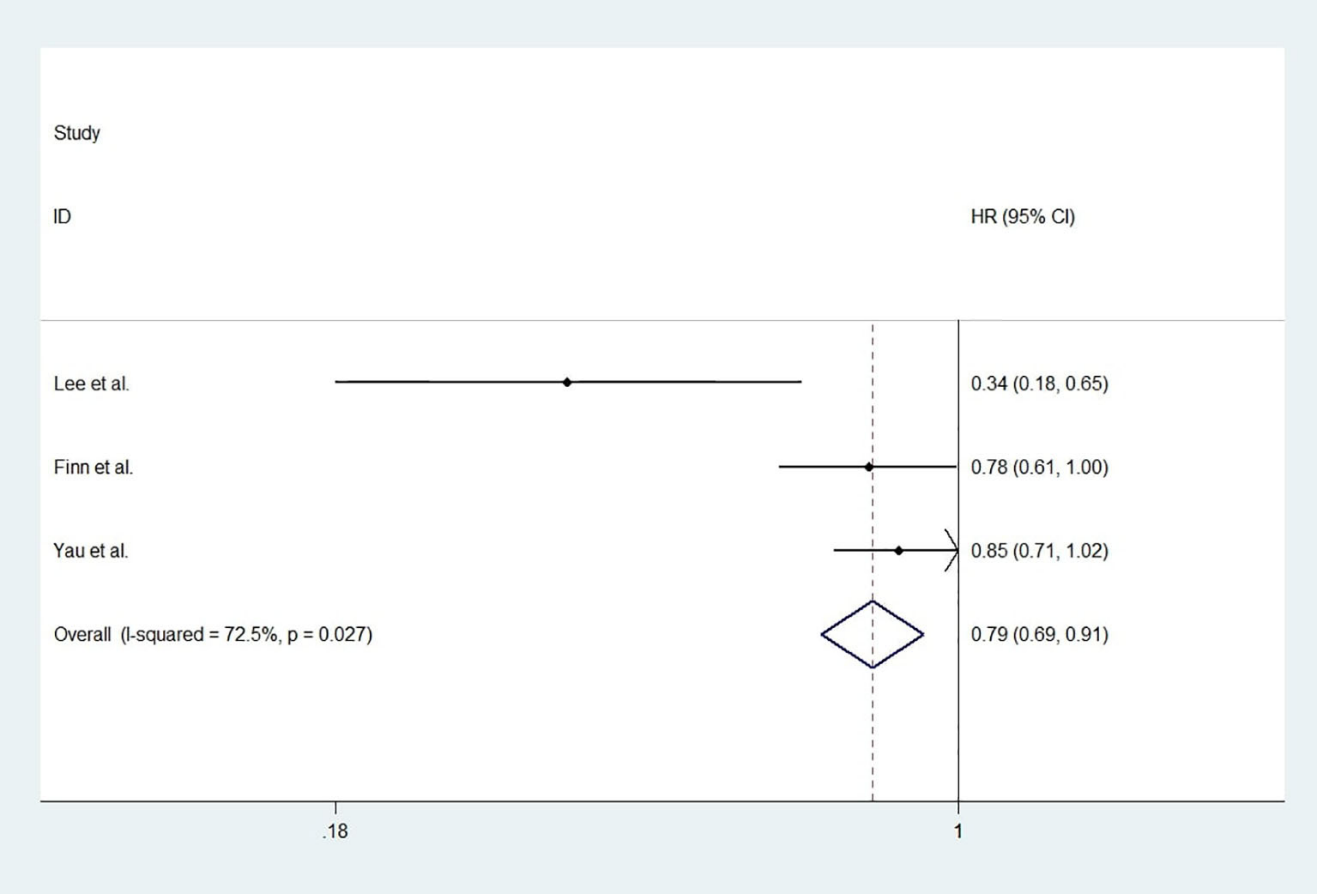
The forest plot of overall survival (OS).

**Table 3:** Treatment-related adverse events in the included studies.

	Any grade	Grade≥3
AST increase	148	61
ALT increase	116	27
Blood bilirubin increased	91	26
Fatigue	89	12
Pruritus	82	1
Diarrhea	71	4
Decreased appetite	66	4
Rash	61	2
Asthenia	57	0
abdominal pain	53	4
AEs leading to discontinuation	51	43
Nausea	44	2
Anemia	42	13
Dyspnea	33	1
Pyrexia	33	2
Back pain	29	4
Hypothyroidism	26	0
Arthralgia	25	1
Lipase increase	23	11
Increased gamma-glutamyltransferase	16	6
Treatment-related deaths	9	0
Myalgia	9	2
Amylase increase	9	2
Hyperbilirubinemia	8	2
Adrenal insufficiency	4	2
Mucosal inflammation	4	1
Hyponatremia	4	3
Cardiac failure	3	1
autoimmune hepatitis	3	2
Gastric ulcer	2	1
Hyperlipasemia	2	1
Iron deficiency anemia	2	1
Lung infection	2	1
Hepatic vein thrombosis	1	1

Some Treatment-related adverse events were not reported in some studies.

AE, adverse events; AST, aspartate aminotransferase; ALT, alanine aminotransferase.

## Discussion

In recent years, the inhibition of PD-1 and PD-L1 pathway has emerged as one of the most potential therapeutic strategies in a variety of cancers, such as melanoma, lung cancer, renal cell carcinoma, head and neck squamous cell carcinoma, etc. (20). This meta-analysis analyzed the existing studies on the treatment of HCC with PD-1 or PD-L1 inhibitors. The results showed that for patients treated by PD-1 or PD-L1 inhibitors, RR, DCR, PFS, OS were 0.17 (0.15-0.19), 0.58 (0.55-0.61), 3.27 months (2.99-3.55), 11.73 months (10.79-12.67), respectively. Compared to the control group, treatment with ICIs significantly improved RR, PFS and OS, the OR and HRs were 3.11 (2.17-4.44, P<0.001), 0.852 (0.745-0.974, P=0.019) and 0.790 (0.685-0.911, P=0.001), respectively. However, no significant improvement in DCR was found in ICIs treatment in this meta-analysis, which may be due to the small number of RCTs or cohort studies included in this study.

Although immunotherapy has achieved certain results, the efficacy of treating some patients with ICI single drug is not ideal. Therefore, similarly to the treatment strategies that were commonly used against other malignant tumors, researchers are now exploring the use of a combination of immune checkpoint inhibitors with other treatments for HCC therapy (21). For example, Finn et al. combined pembrolizumab (a PD-1 inhibitor) with lenvatinib (a multikinase inhibitor) to treat unresectable HCC (uHCC), and found that lenvatinib plus pembrolizumab has promising antitumor activity in uHCC. Toxicities were manageable, with no unexpected safety signals (22). Xu et al. used apatinib and SHR-1210 (camrelizumab) to treat advanced HCC and results also showed manageable toxicity in patients with HCC (23). Other combinations include ICIs combination, ICIs combined with MTA (molecular targeted agents), ICIs combined with local/systemic therapy (21).

However, immunotherapy, as a drug class, boosts the body’s natural defense against cancer. These drugs have adverse effects, collectively known as immune-related adverse events, that represent immune effects on normal tissue that can result from misdirected stimulation of the immune system (24). There were also a lot of adverse events reported in the included studies, such as infection, rash, pruritus, reactive cutaneous capillary endothelial proliferation (RCCEP), increased aspartate aminotransferase (2, 6, 16).

There were also many deficiencies in this meta-analysis. Firstly, although 12 studies were finally included in this study, 8 of them were single-arm studies and only 2 were RCTs, and the lack of comparison data made it difficult to provide solid evidence of the efficacy and safety in the treatment of HCC with ICIs. Most of the studies included in this meta-analysis were single-arm studies, which only provided information on patients treated with ICIs, but did not have a control group for comparison. The direct merging of the single-arm studies data with the comparative studies data in the article might make the results of this meta-analysis unstable. At the same time, it was very difficult for us to perform the bias analysis due to the lack of studies with comparison data in this meta-analysis. Secondly, all the ICIs used in the included studies were PD-1 inhibitors (nivolumab, pembrolizumab, camrelizumab, cemiplimab and tislelizumab), so that the efficacy and safety of PD-L1 inhibitors in the treatment of HCC could not be analyzed, such as atezolizumab, durvalumab et al. Besides, the classification of HCC, the dosage and method of administration of ICIs were not identical, which could affect the reliability of the meta-analysis results. In general, there were still few studies on the treatment of HCC with PD-1 or PD-L1 inhibitors, especially the high-quality RCT studies that would reveal the efficacy and safety of PD-1 or PD-L1 inhibitors in the treatment of HCC. The good news is that there are a lot of studies going on right now, such as KEYNOTE-394 (25) (pembrolizumab plus best supportive care vs. placebo plus best supportive care), RATIONALE-301 (26) (tislelizumab vs sorafenib), etc. It is hoped that in the future, more objective and rich data can be obtained from these studies, so as to provide more useful evidence for the treatment of HCC by PD-1 or PD-L1 inhibitors.

## Data Availability Statement

The original contributions presented in the study are included in the article/supplementary material. Further inquiries can be directed to the corresponding author.

## Author Contributions

XW conceived the idea for the study and assessed the quality of the manuscript. KF was responsible for data acquisition. SH and WJ performed the meta-analysis and co-drafted the manuscript. All authors contributed to the article and approved the submitted version.

## Funding

National Natural Science Foundation of China (NO. 81501828).

## Conflict of Interest

The authors declare that the research was conducted in the absence of any commercial or financial relationships that could be construed as a potential conflict of interest.
